# Stimulus rate increases lateralisation in linguistic and non-linguistic tasks measured by functional transcranial Doppler sonography

**DOI:** 10.1016/j.neuropsychologia.2015.04.019

**Published:** 2015-04-20

**Authors:** Heather Payne, Eva Gutierrez-Sigut, Joanna Subik, Bencie Woll, Mairéad MacSweeney

**Affiliations:** a Deafness, Cognition & Language Research Centre, University College London, 49 Gordon Square, London WC1H 0PD, United Kingdom; b Institute of Cognitive Neuroscience, University College London, 17 Queen Square, London WC1N 3AR, United Kingdom

**Keywords:** fTCD, Hemispheric lateralisation, Language, Rhyme judgement, Task difficulty, Visuospatial, Line judgement

## Abstract

Studies to date that have used fTCD to examine language lateralisation have predominantly used word or sentence generation tasks. Here we sought to further assess the sensitivity of fTCD to language lateralisation by using a metalinguistic task which does not involve novel speech generation: rhyme judgement in response to written words. Line array judgement was included as a non-linguistic visuospatial task to examine the relative strength of left and right hemisphere lateralisation within the same individuals when output requirements of the tasks are matched. These externally paced tasks allowed us to manipulate the number of stimuli presented to participants and thus assess the influence of pace on the strength of lateralisation.

In Experiment 1, 28 right-handed adults participated in rhyme and line array judgement tasks and showed reliable left and right lateralisation at the group level for each task, respectively. In Experiment 2 we increased the pace of the tasks, presenting more stimuli per trial. We measured laterality indices (LIs) from 18 participants who performed both linguistic and non-linguistic judgement tasks during the original ‘slow’ presentation rate (5 judgements per trial) and a fast presentation rate (10 judgements per trial). The increase in pace led to increased strength of lateralisation in both the rhyme and line conditions.

Our results demonstrate for the first time that fTCD is sensitive to the left lateralised processes involved in metalinguistic judgements. Our data also suggest that changes in the strength of language lateralisation, as measured by fTCD, are not driven by articulatory demands alone. The current results suggest that at least one aspect of task difficulty, the pace of stimulus presentation, influences the strength of lateralisation during both linguistic and non-linguistic tasks.

## 1 Introduction

Functional transcranial Doppler sonography (fTCD) uses ultra-sound to measure changes in the speed of blood flow through the left and right middle cerebral arteries (MCAs) during the performance of sensory and cognitive tasks (Aaslid et al., 1982). Studies using this technique have reported a comparable extent of left hemisphere dominance during language tasks as fMRI (Deppe et al., 2000; Somers et al., 2011) and the gold standard test of language lateralisation, the Wada procedure (Knake et al., 2003; Knecht et al., 1998). Concordance between fTCD and fMRI is also reported for right hemisphere dominance during spatial attention tasks (Jansen et al., 2004). These studies provide good validation of the use of fTCD to measure hemispheric dominance of cognitive function, despite differences in the physiological markers measured by different neuroimaging modalities.

fTCD offers a relatively cheap, easy and non-invasive way to assess hemispheric dominance during cognitive tasks. Recently, it has been used to investigate the development of language lateralisation in young children and special populations (Chilosi et al., 2014; Groen et al., 2012). To date, the primary experimental task used has been word generation (e.g. verbal fluency as in Deppe et al. (2000) and Knecht et al. (1998)) or with children, sentence generation in the form of picture or video description (Lohmann et al., 2005; Bishop et all., 2009; Haag et al., 2010; Groen et al., 2012; Chilosi et al., 2014). These studies converge with findings from other neuroimaging modalities indicating a robust and pervasive leftward asymmetry in functional responses during expressive language production. In order to maximise the contribution of fTCD to the field, and to further our understanding of developmental changes in language lateralisation it would be beneficial to take a multidimensional approach to language (Bishop, 2013) by examining language lateralisation across a range of different language skills and not only during generation of novel material.

During free generation tasks such as verbal fluency, participants are required to think of or articulate as many words as possible, leading to considerable inter- and intra-individual variability in the amount of subvocally generated or overtly articulated words. We speculate that this variability contributes to individual differences in the degree of lateralisation that is measured. Results from our recent study suggest this may be the case (Gutierrez-Sigut et al., 2015). Strength of lateralisation was positively correlated with the number of words produced, suggesting a relationship between the signal measured using fTCD and the premotor requirements of the task.

Our primary question in the current study was whether language lateralisation could be robustly measured using fTCD during a metalinguistic judgement task, which permits a level of control of the amount of articulatory planning required. To achieve this we used a written word rhyme judgement task, which does not require mental generation of novel items, but, we reasoned, still sufficiently engages articulatory planning processes. During rhyme judgement of orthographically dissimilar word pairs, participants must sub-vocally rehearse items in order to correctly complete the task. The choice of a rhyme judgement task was also motivated by fMRI studies reporting peaks in activation during rhyme judgement in left posterior mid and inferior prefrontal gyri (Booth et al., 2002; Kareken et al., 2000; Lurito et al., 2000; Paulesu et al., 1997; Xu et al., 2001). These are regions perfused by the middle cerebral artery (MCA), from which measurements are made using fTCD.

A second aim of the study was to examine how ‘linguistic’ and ‘non-linguistic’ tasks affect the fTCD signal within participants. Previous studies have also examined this, with the aim of testing the nature of the relationship between hemispheric specialisation across cognitive domains. It is interesting that these studies used the standard word generation task as the ‘linguistic’ task and either a visual memory (Lust et al., 2011; Whitehouse and Bishop, 2009), spatial orientation (Dorst et al., 2008) or a line bisection task (Flöel et al., 2005; Badzakova-Trajkov et al., 2010; Rosch et al., 2012) as the ‘non-linguistic’ task. Whilst this approach has made important contributions to the field, it presupposes that the tasks being used are equally representative exemplars of a whole cognitive domain i.e. verbal or visuo-spatial (here we use linguistic and non-linguistic for consistency). An alternative view is that these linguistic and non-linguistic tasks have very different processing and output demands. For example, differences in the format of visual stimuli (e.g., videos versus single letters) may influence blood flow to a greater extent than the domain being tested (linguistic or non-linguistic).

Here, we examine the variability of hemispheric lateralisation for linguistic and non-linguistic processing, using paced judgement tasks which were well matched in terms of task demands: rhyme judgement in response to written word pairs and line similarity judgement in response to visual line arrays. Again, the choice of non-linguistic task was informed by the fMRI literature. Kareken et al. (2000) asked participants to make same/different judgements to line arrays in addition to rhyme judgements to orthographically dissimilar rhyme pairs. They reported greater left than right hemisphere activation for the rhyme task. In the line judgement task, they reported strongly right lateralized activation over a large proportion of the posterior parietal lobe, and a distinct area in the right posterior middle temporal gyrus, an area supplied by the MCA.

One benefit of using externally paced judgement tasks is that it allows the direct manipulation of task demands via the number of stimuli presented. The final aim of the study was to characterise the influence of task demands on language lateralisation. Though ‘task demands’ can refer to a variety of different factors, in the current study we address one specific element, that of pace, by increasing the number of judgements to be made during the active period. We predict that increasing the pace of judgements required will lead to increased strength of lateralisation. During the rhyme judgement task, two factors are hypothesised to drive this increase ȓ the greater number of words to subvocally articulate (placing higher demands on premotor processes) and the increased cognitive effort of completing the task at a faster pace.

Previous studies that have examined the relationship between the number of words articulated and strength of LI have typically reported low or non-significant correlations (e.g. Knecht et al., 2000). However, in these studies the amount produced has been inferred from the overt report period following covert generation. In contrast, we have shown that the amount of material generated and strength of LI do correlate positively when concurrent measures are taken during an overt word generation task (Gutierrez-Sigut et al., 2015).

Studies that have manipulated cognitive effort have done so via the familiarity of the stimulus, with no control over the output. For example, Dräger and colleagues conducted covert word retrieval tasks with fMRI (Dräger et al., 2004) and fTCD (Dräger and Knecht, 2002). Difficulty was manipulated by presenting word stems of high and low frequency and instructing participants to covertly retrieve legal words using the target stems. There were no differences in the strength of lateralisation between high and low frequency stimuli, either in the fMRI or fTCD data. Using a similar approach, Badcock et al. (2011) manipulated task difficulty using letters of greater or lesser frequency in a covert word generation task. They reported no differences in lateralisation between difficulty levels. Here task difficulty was categorised into low, medium, and high productivity letters, based on the average number of reported words after the active period. As suggested above, however, this method is a somewhat indirect measure of amount produced during the covert period, and therefore also of difficulty.

Here we predict that an increase in the rate of presentation will lead to an increase in the strength of left lateralisation during the rhyme judgement task, due to the combined factors of a greater number of words to subvocally rehearse and increased task difficulty. By testing the effect of pace on a non-linguistic task, we go some way to tease apart these factors. A finding of stronger lateralisation in fast paced conditions for both rhyme and line tasks implies task difficulty associated with increased pace, rather than articulatory planning demands being the sole driver of the strength of lateralisation.

In summary, in Experiment 1 we tested whether left and right lateralisation can be established using fTCD during rhyme and line array judgement tasks which were well matched in their demands. In Experiment 2 we sought to determine the effect of pace on lateralisation for linguistic and non-linguistic tasks, by manipulating the number of stimuli presented during a trial.

## 2 Experiment 1

### 2.1 Method

#### 2.1.1 Participants

A total of 38 right-handed participants were recruited for Experiment 1. All participants were monolingual native speakers of British English. No participants reported a history of neurological disorders or language related problems. Participants were all right handed as assessed by an abridged version of the Edinburgh Handedness Inventory (Oldfield, 1971). To screen for reading difficulties which are associated with impaired metalinguistic abilities (Wimmer et al., 1994), reading comprehension was assessed using the Kirklees Reading Assessment (Vernon-Warden revised; Hedderly, 1993).

Data from several participants were excluded because of inability to find a signal or poor signal quality (6 cases), low reading comprehension scores (greater than 2 sd below the group average; 2 cases), and/or low accuracy on the experimental tasks (scores lower than 2 sd below the group mean ( < 83% on rhyme or < 81% on line; 2 cases). Therefore data from 28 (11 male) participants were included in the study. The average age of participants was 26.2 years (sd 6.4; range: 18.60–49.56). The average reading score was 34.66 (sd 3.48; range 27–40 max=42), which corresponds to a mean reading level categorised as ‘adult’ on the test used (range: 16 years to 23 years + ). Of the 28 participants, 21 were students at UCL and 7 were from the local community. These participants did not differ in age (*t*(7.17)=1.6, *p*=.15) or reading score (*t*(8.53)=.25, *p*=.80 (analyses adjusted for unequal variances using Welch–Satterthwaite adjusted *t*-tests).

#### 2.1.2 Stimuli

##### 2.1.2.1 Rhyme judgement stimuli

Rhyme stimuli were 180 words presented in 90 word pairs (based on those in MacSweeney et al. (2013)). Half of the word pairs rhymed and half did not (see Table 1 for examples). All words were monosyllables and had a single coda. To ensure that the rhyme decision could not be made on the basis of spelling similarity (orthography) of the items in a pair, the orthographic similarity of word pairs was measured using the metric of Davis (2010) (http://www.pc.rhul.ac.uk/staff/c.davis/Utilities/MatchCalc/). This metric takes into account letter position to estimate the overall orthographic similarity between two words: a value of ‘0’ indicates no overlap and ‘1’ indicates identical letter strings. The mean overlap values were: rhyming word pairs=.34 (sd=.13), non-rhyming word pairs=.33 (sd=.13). There was no significant difference between word sets (*t*(88)=.65, *p*=.94, cohen's *d*=.01). On average, the rhyming and non-rhyming sets were also matched on number of letters, number of phonemes, frequency (Francis and Kucera, 1982), and, where data were available, from the MRC database (Coltheart, 1981) on number of orthographic neighbours, familiarity, concreteness and imageability (all *p*s > .1).

##### 2.1.2.2 Line judgement stimuli

**Fig. 1:**
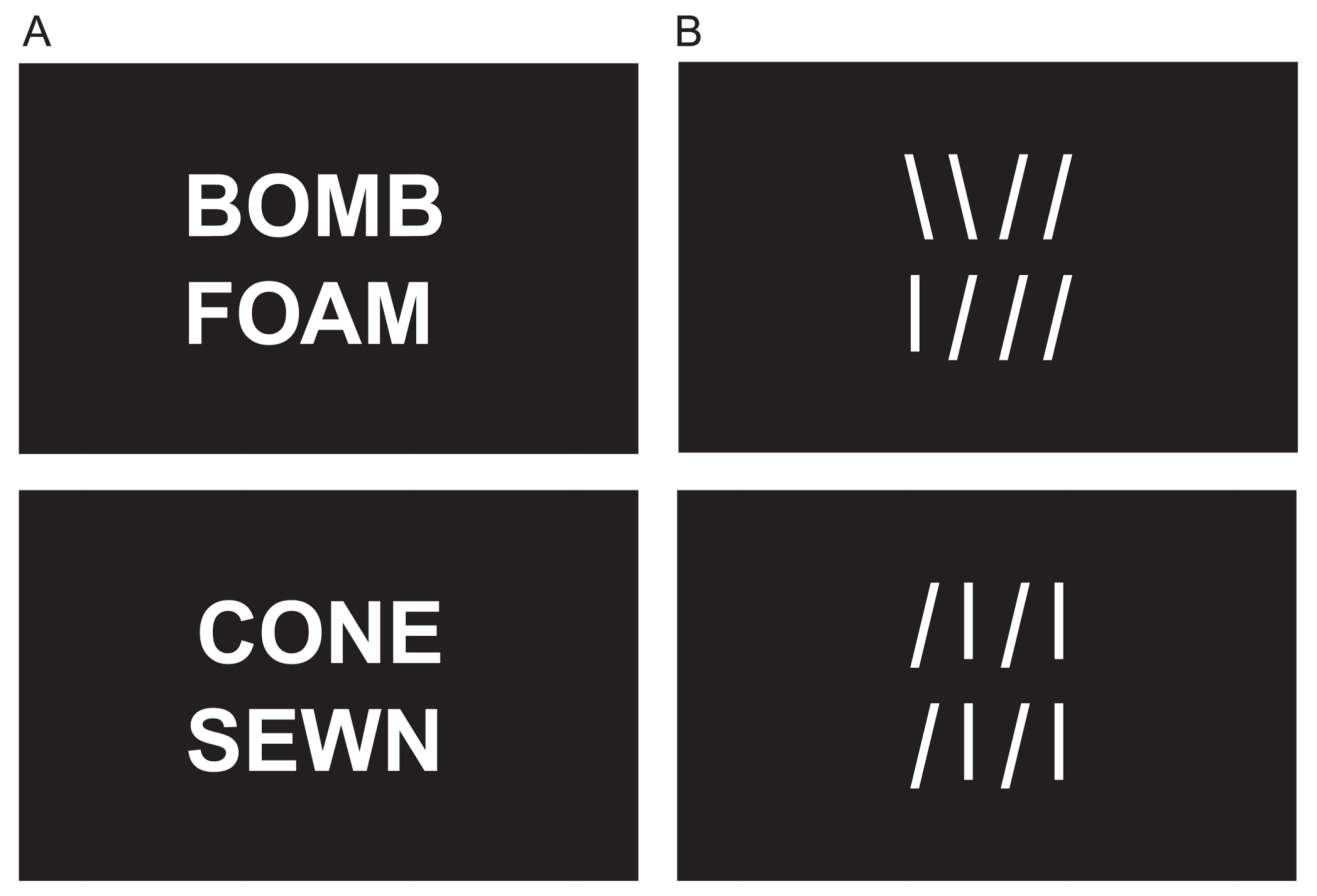
Examples of the presentation format for (A) rhyming and non-rhyming word pairs, (B) matching and non-matching line sets.

Stimuli were 180 line sets presented in 90 pairs, one item above the other (see Fig. 1). Line sets comprised a series of 3–6 vertical and angled lines. The number of lines in each array was matched to the number of letters in the rhyming words. Half of the line array pairs were identical and half were dissimilar by one or two line orientations. Line sets were created from text characters in the same point size as letters. Behavioural piloting showed comparable accuracy and reaction times for word and line stimuli.

#### 2.1.3 Procedure

Participants were seated facing a laptop computer upon which time-locked stimuli were presented using Psychtoolbox-3 (Brainard, 1997; Kleiner et al., 2007) for MATLAB 2012b (Mathworks Inc., Sherborn, MA, USA). Triggers were sent from the presentation computer via parallel port to the Doppler-Box set-up at trial onsets and recorded on a separate data acquisition computer with the TCD signal, allowing the analysis of stimuli-related changes in cerebral blood flow.

**Fig. 2:**
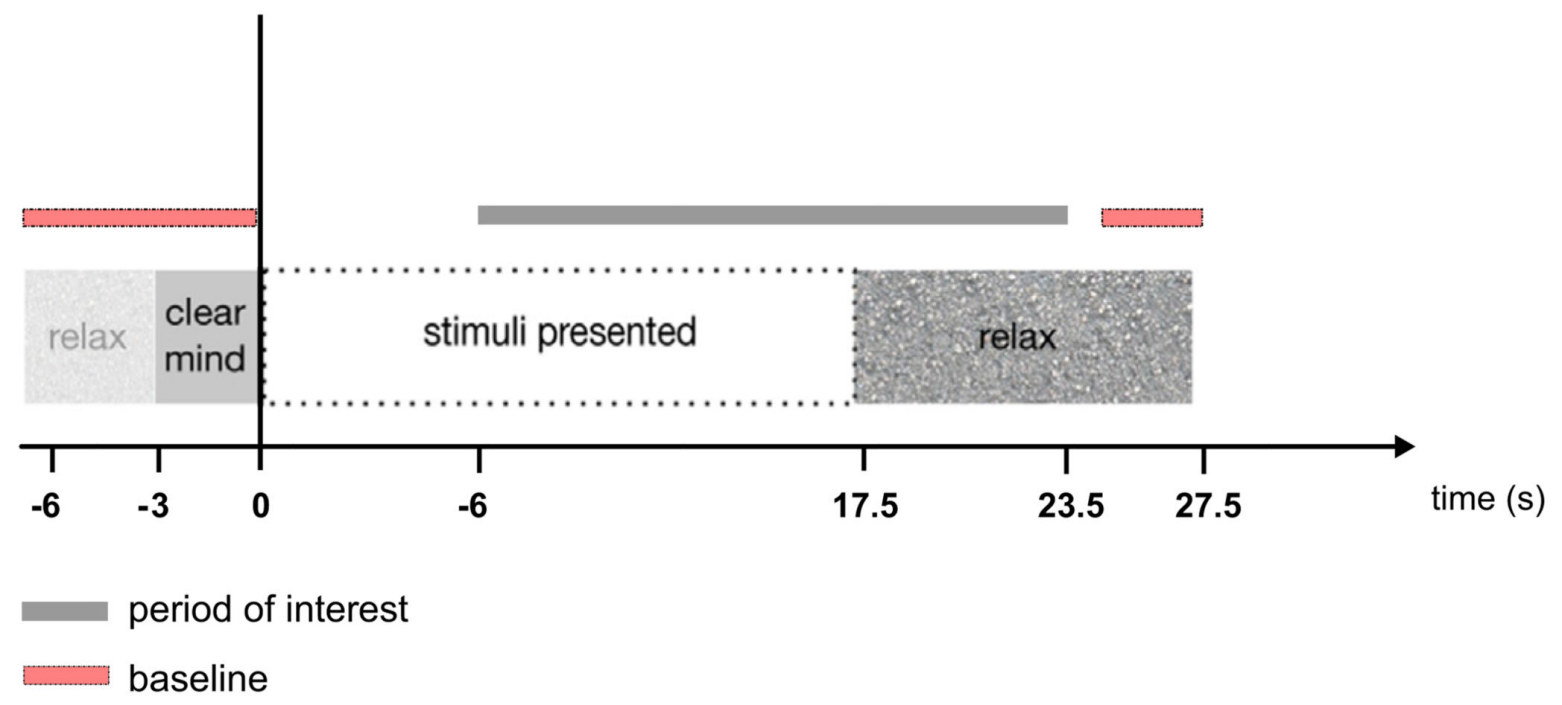
Schematic of the timing of events for rhyme and line judgement tasks in Experiment 1.

Participants performed both rhyme and line judgement tasks. The order of the tasks was counterbalanced across participants. Trials began with a three second ‘clear mind’ period, during which participants were instructed to focus on the black of the screen. This was followed by the presentation of five successive stimulus pairs (either words or lines). Participants had to judge whether word pairs rhymed or line arrays were the same or different. Each active phase lasted for 17.5 s. After the active phase there was a 10 s ‘relax’ period in which participants were instructed to imagine a visual scene. We have previously used this duration of relax period to allow normalisation of the blood flow to baseline (Gutierrez-Sigut et al., 2015). The whole test cycle for each trial was 30.5 s and there were 18 trials for each condition (see Fig. 2). The rhyme and line judgement tasks were performed in separate blocks, each lasting 9 min, 9 s.

Button press ‘yes’ (rhyme/ matching lines) and ‘no’ (non-rhyme/ non-matching lines) responses were made with the index fingers of each hand. Participants were instructed to keep their index fingers in a comfortable position over the keys to minimise movement. The button indicating match or mismatch was counterbalanced across participants but was kept consistent for the participant across tasks. The keys ‘Z’ and ‘M’, as found on a typical QWERTY keyboard, were used to record responses. Accuracy and reaction time data were recorded for each item. Both ‘yes’ and ‘no’ trials were presented within each epoch. However, since fTCD is measuring a haemodynamic signal, it has relatively poor temporal resolution and therefore it is currently not possible to disambiguate blood flow responses to rhyme versus non-rhyme, or line match versus line mismatch, trials in the fTCD signal.

#### 2.1.4 Data analysis

Data were analysed using a custom toolbox for MATLAB, dopOSCCI (Badcock et al., 2012). Artefact rejection thresholds were set such that epochs containing blood flow velocities less than 70% or greater than 130% of the average velocity for that individual were rejected. As is the current standard for fTCD analysis, the maximum left–right difference allowed was set to 20% after normalization (where the mean blood flow velocity for the total sample is adjusted to 100) to further protect from the possibility of inaccurate signals contributing to averages.

Blood flow velocity changes were analysed on a trial-by-trial basis from −6 to 23.5 s post-initial stimulus presentation. The sample points measured from each artery were corrected to a pre-stimulus baseline period from −6 to 0 s, to protect against differences across trials in the low frequency components of cerebral blood flow. A period of at least 10 s of recording was made before the start of the first trial to allow a baseline for the first trial. Participants fixated on the screen for this time.

Strength of differences between blood flow responses in left and right MCAs are most often quantified using Laterality Indices (LIs). To calculate these, periods of interest (POIs) were set from 6 to 23.5 s to allow for a lag in the blood flow speed response poststimulus. Within this window the maximum difference in blood flow between left and right was identified. Laterality Indices for each individual are given by the mean difference between left and right over a 2 s interval around this peak. This is the current standard method for analysing fTCD data (see Badcock et al., 2012; Deppe et al., 2004).

### 2.2 Results

#### 2.2.1 Behavioural data

Table 2 shows accuracy and reaction time data for the rhyme and line judgement tasks. Paired *t*-tests showed no significant difference in accuracy between the tasks (*t*(27)=.78, *p*=.44 cohen's *d*_z_=.15); however reaction times during the line judgement task were significantly longer than during rhyme judgement (*t* (27)=4.21, *p*=< .001, cohen's *d*_z_=.80).

#### 2.2.2 fTCD data

##### 2.2.2.1 Artefact rejection and reliability

After artefact rejection there were a comparable number of trials for rhyme and line tasks (*t*(27)=.35, *p*=.7 cohen's *d*_z_=.06 rhyme mean=17.1 (sd 1.1), line mean=17.0 (sd 1.1). All participants had at least 14 acceptable trials (min=14, max=18). To assess reliability, we conducted split half correlations between LIs from odd and even trials. The rhyme task showed good split half reliability: (*r*=.55, *p*=.002). The line task was less consistent, showing a moderate correlation approaching significance (*r*=.34, *p*=.06).

##### 2.2.2.2 Group analyses

Group mean and median LIs for the rhyme and line judgement tasks are shown in Table 3. Rhyme and line tasks showed group level left and right lateralisation respectively in 1 sample *t*-tests (rhyme: *t*(27)=2.48, *p*=.02, cohen's *d*_z_=.46; line: *t*(27)=4.44, *p*=< .001, cohen's *d*_z_=.84).

The majority of fTCD studies categorise individuals into ‘left’, ‘right’ and ‘low’ (or ‘bilateral’) laterality based on the extent and direction of their lateralisation index. An individual's standard error is used to determine whether they are significantly different from zero, which indicates equal blood flow change in left and right MCAs. The categorisation of participants in this way is also shown in Table 3.

We tested whether the strength of lateralisation was significantly different for the two tasks with a *t*-test on the rhyme LIs with reversed sign for the line LIs. This was non-significant (*t*(27)=1.55, *p*=.13, cohen's *d*_z_=.29) implying comparable strength of lateralisation in each task. However, there was no evidence for a correlation between strength of lateralisation on the rhyme and line judgement tasks (*r*=.06, *p*=.77).

### 2.3 Summary of Experiment 1

In Experiment 1, 28 right-handed participants showed group level left hemisphere lateralisation, as measured using fTCD, when performing a metalinguistic task that does not require overt or covert word generation. Furthermore, right hemisphere lateralisation was also established for a non-linguistic task, which was matched to the linguistic (rhyme) condition in task requirements. This suggests that fTCD is indeed sensitive to ‘verbal’ and ‘nonverbal’ processing, above and beyond the cognitive requirements of completing a match/mismatch decision.

We note that the group mean LI of .84 during the rhyme judgement is lower than those LIs reported in previous studies of word generation (e.g. 2.7 (Stroobant et al., 2009); 1.69 (Bishop et al., 2009); 2.11 (Somers et al., 2011); 3.19 (Krach et al., 2006); 3.94 (Dorst et al., 2008); 2.41 (Badcock et al., 2011). In addition, considering the data categorically, we find a lower percentage of participants categorised as significantly left lateralised (36%) than previously reported (e.g. 82% (Bishop et al., 2009b); 85% (Flöel et al., 2005)). The proportion of participants categorised as right lateralised for the line judgement task was also low (50%) compared to previous studies of right-handed adults: for example, 75% (whitehouse and Bishop, 2009) and 72% (Dorst et al., 2008). It is possible that the low lateralisation in Experiment 1 is due to the slow pace of stimulus presentation. Given our previously reported association between strength of lateralisation and number of words generated (Gutierrez-Sigut et al., 2015), we reasoned that making more rhyme judgements in the same period could boost premotor activity and result in higher LIs measured using fTCD.

To test the hypothesis that an increase in pace would lead to an increase in strength of left hemisphere dominance, we contrasted performance on slow and fast paced rhyme judgement tasks in a within subjects design. We predicted that an increase in the rate of presentation would lead to an increase in the strength of left lateralisation during the rhyme judgement task. We hypothesised this to be due to both the increased in the amount of material to be sub-vocally rehearsed and the increase in task difficulty resulting in greater effort. These factors can be teased apart to some extent by testing the effect of pace on a non-linguistic task.

## 3 Experiment 2

### 3.1 Method

#### 3.1.1 Participants

Eighteen of the participants who performed Experiment 1, also performed fast paced versions of the judgement tasks. However, to enable the data from Experiment 1 and Experiment 2 to be contrasted directly, steps were taken to avoid practice and order effects. All participants who had already taken part in Experiment 1 were invited back to take part in Experiment 2. Nine participants (6 male) responded and subsequently performed the fast paced version of the tasks (Experiment 2). The remaining 9 cases were first recruited to perform Experiment 2 and returned at a later date to perform Experiment 1.

The mean age of these participants was 26.9 years (sd=7.1). Performance of the fast and slow paced tasks was counterbalanced and each participant (except one) performed the two levels of pace in separate sessions. All participants were right-handed and the average reading score (Kirklees Reading Assessment, Vernon-Warden revised; Hedderly, 1993) was 34.5 (sd=4.09), which corresponds to a reading level categorised as ‘adult’.

#### 3.1.2 Stimuli and procedure

Stimuli for the fast paced versions of rhyme and line judgement tasks were the same as for the slow paced version (see Section 2.1.2) but each pair was presented twice throughout the session, in a pseudorandomised order. Trials proceeded in the same way for the slow paced and fast version, with the exception of the number of items presented in the active period. Ten stimuli, each displayed for 2.1 s, were presented in each epoch of the fast paced version. This is in contrast to the presentation of five stimulus pairs for 3.5 s each in Experiment 1 (see Fig. 2). Therefore, the active period for the fast paced condition was 21 s, compared to 17.5 s in the slow paced version. The longer period was necessary to allow all of the stimuli to be presented twice at the fast presentation rate, but maintaining the same number of trials as in Experiment 1. Faster stimulus presentation was not possible since piloting established that presenting the line stimuli for less than 2.1 seconds would have led to a considerably higher error rate.

#### 3.1.3 Data analysis

Artefact rejection thresholds and baseline correction parameters were the same as for Experiment 1 (see Section 2.1.4). It could be argued that a more appropriate length of epoch for the fast paced condition is −6 to 27.5 s, to account for the longer stimulus presentation period. The analyses were rerun with this longer epoch length and this did not affect the outcomes reported here. It seems therefore likely that the marginally longer presentation period did not affect the physiological responses to the stimuli in a way which would bias left–right blood flow responses. As in Experiment 1, epochs were analysed from −6 s to 23.5 s post-initial stimulus. Periods of interest (POIs) were set from 6 to 23.5 s. Data were analysed using IBM SPSS 21 using the GLM Repeated Measures procedure, to control for non-independency of the LIs. We used a 2 × 2 full-factorial design with pace (fast versus slow) and task (rhyme versus line) as within-subject factors.

### 3.2 Results

#### 3.2.1 Behavioural data

**Fig. 3:**
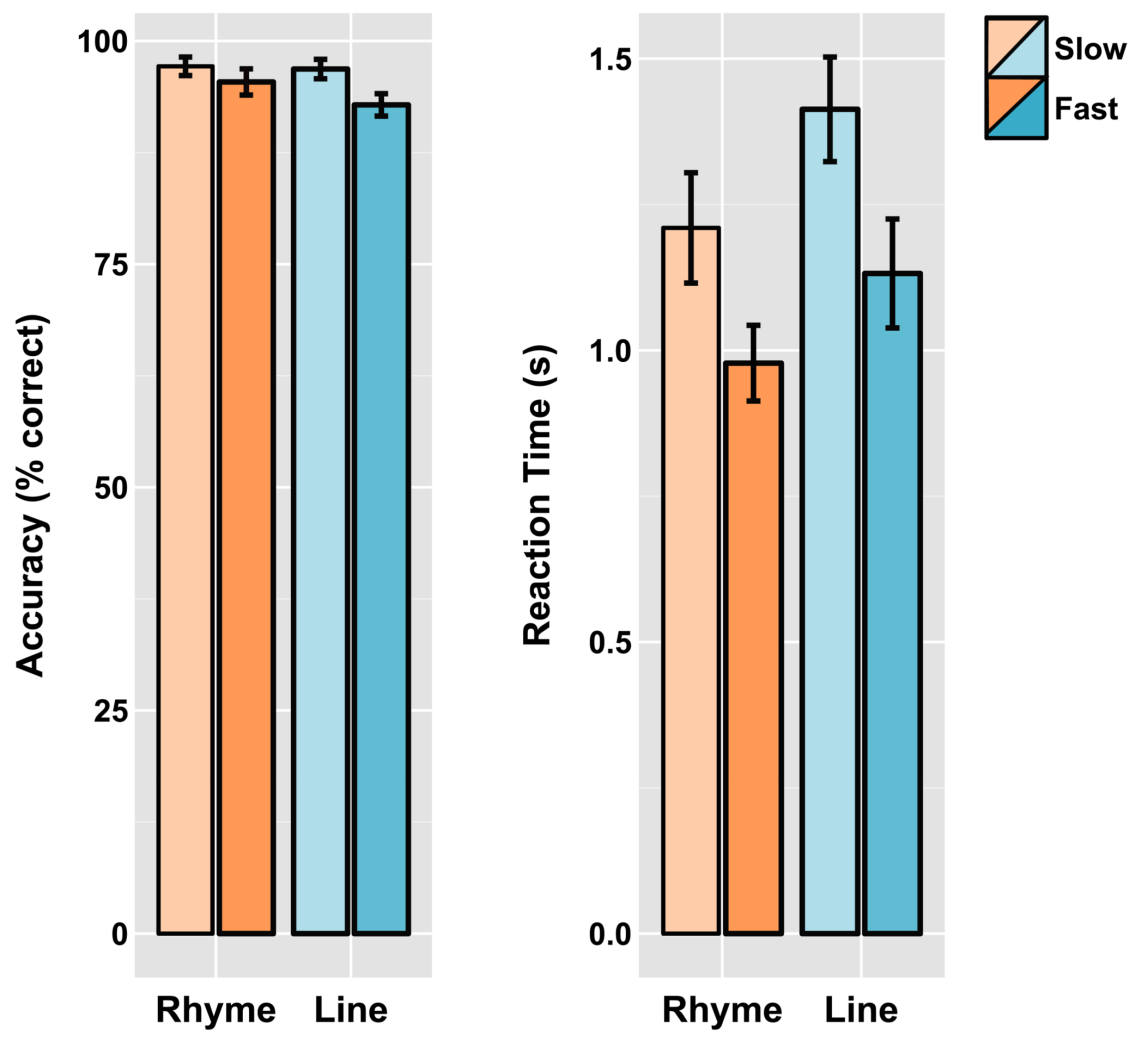
Mean accuracy and reaction time summaries for rhyme and line judgement at each level of presentation speed in Experiment 2.

Mean accuracy and reaction time data for the four conditions are plotted in Fig. 3. Data from 2 participants were lost due to technical problems during recording. Therefore data from 16 participants are reported. A 2 (fast versus slow) x2 (rhyme versus line) ANOVA on the accuracy data showed a main effect of task (*F*(15)=8.76, *p*=.01, MSE=3.71), this was due to a higher level of accuracy on the rhyme task than the line task. There was also a significant main effect of pace (*F*(15)=16.97, *p*=.001, MSE=9.08) indicating higher accuracy in the slow compared to fast condition. There was also a significant interaction between task and pace (*F*(15)=5.13, *p*=.04, MSE=4.12). The interaction was due to the fact that the faster pace of presentation led to a greater drop in performance in the line condition (*t*(15)=4.92, *p*=<.001 cohen's *d*_z_=.31), than in the rhyme condition (*t*(15)=2.06, *p*=.06, cohen's *d*_z_=.13).

The same analysis of the reaction time data showed a main effect of task (*F*(15)=13.79, *p*=.002, MSE=.039), indicating longer reaction times to line judgements than rhyme judgements and the expected main effect of pace (*F*(15)=36.03, *p*<.001, MSE.03) indicating faster reaction times to the fast paced than slow paced stimulus presentation. This is expected given the fast paced stimuli were displayed for a shorter amount of time. The interaction was not significant (*F*(15)=.86, *p*=.38, MSE=.02).

#### 3.2.2 fTCD data

##### 3.2.2.1 Artefact rejection and reliability

Trial rejection rates due to artefacts were low. There were no differences in the number of accepted epochs between rhyme and line tasks in either slow or fast versions of the task (slow: *t*(17)=.26, *p*=.7, cohen's d_z_=.06, fast: *t*(17)=.25, *p*=.8, cohen's *d*_z_=.19. All participants had at least 14 accepted trials (slow min=14, max=18, fast min=16, max=18). Split half-reliabilities for slow and fast rhyme judgement conditions were good (*r*=.63, *p*=.005 and *r*=.67, *p*=.002). Split half-correlations for slow and fast line judgement revealed lower consistency (*r*=.15, *p*=.55 and *r*=.24, *p*=.33).

To test the consistency between fast and slow speeds, we tested the correlation between LI at each speed, and this was significant for both rhyme (*r*=.60, *p*=.008) and line (*r*=.52, *p*=.028) tasks.

##### 3.2.2.2 Lateralisation indices

**Fig. 4:**
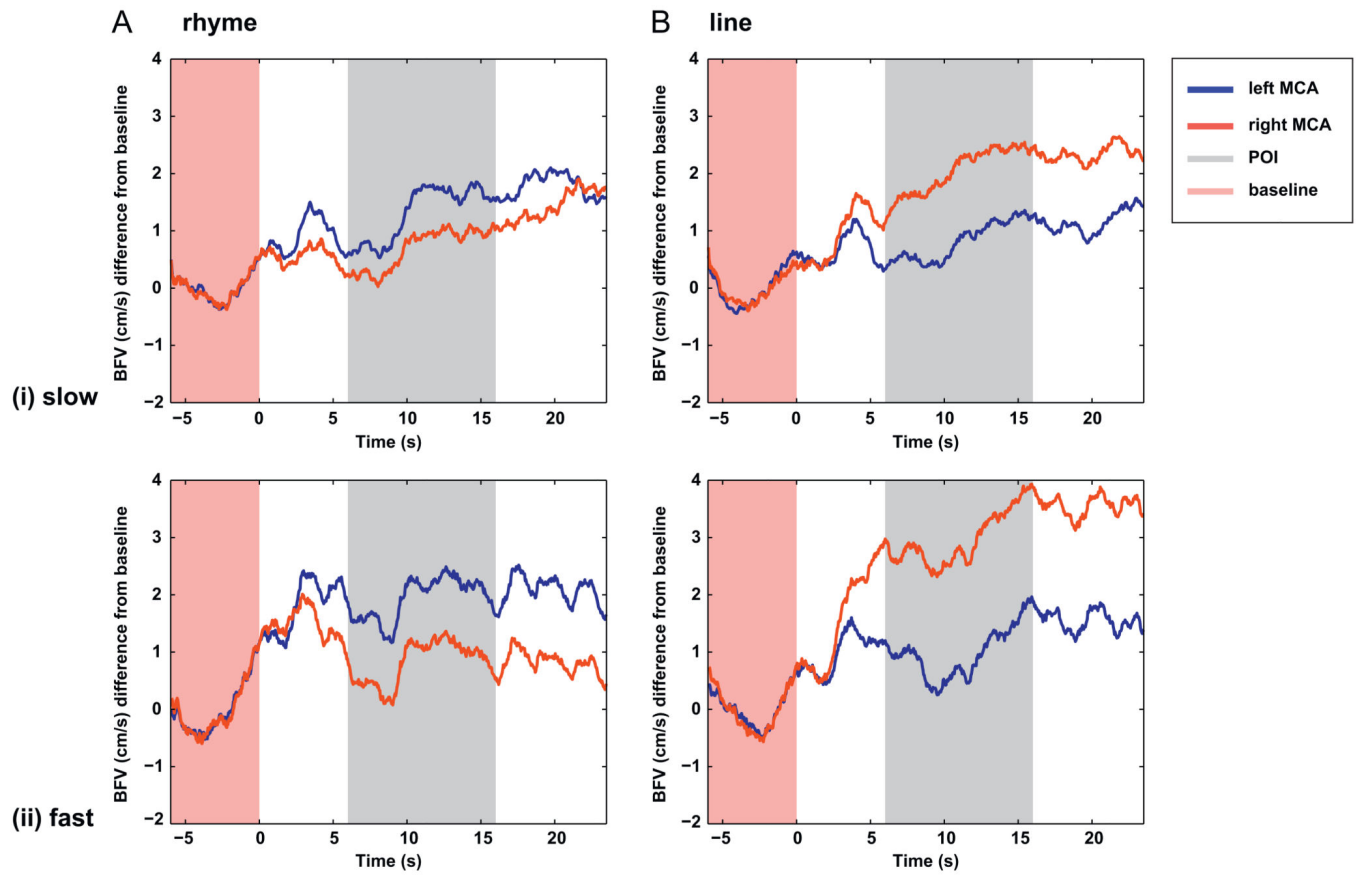
Average of participants' baseline-corrected cerebral blood flow velocity for the left (blue) and right (red) channels for rhyme judgement (Panel A) and line judgement (Panel B). The uppermost plot (i) depicts blood flow velocity change during the original slower paced presentation. The figure beneath (ii) depicts the faster paced presentation. The grey section indicates the period of interest within which the lateralisation indices (LIs) were calculated from the individuals’ maximum left-right difference. (For interpretation of the references to colour in this figure legend, the reader is referred to the web version of this article.)

**Fig. 5:**
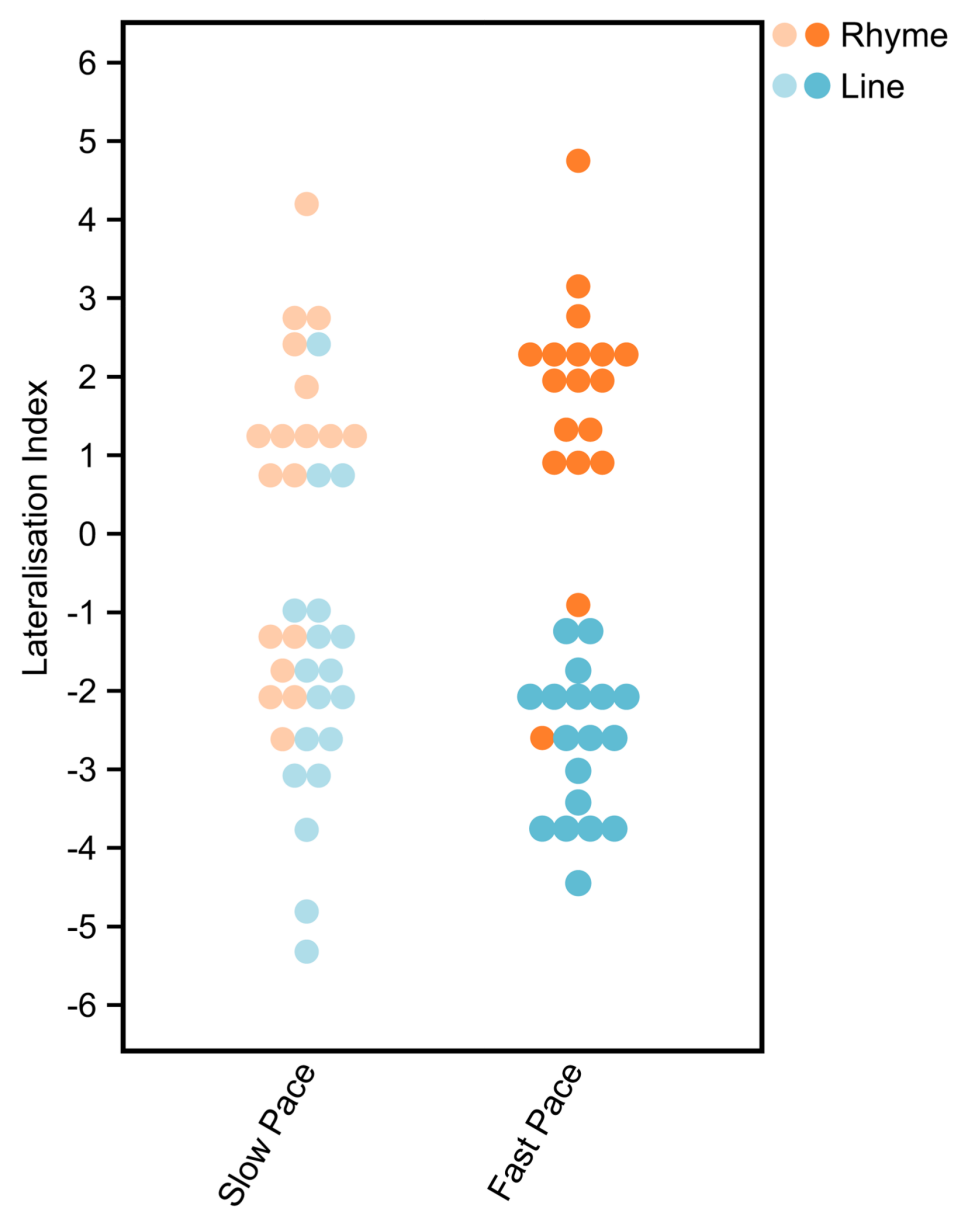
Distribution of individuals' lateralisation indices measured during slow (left) and fast (right) presentation speeds. Positive indices denote greater left than right cerebral blood flow change. Negative values denote greater right than left cerebral blood flow change.

Group mean and median LIs for the rhyme and line judgement tasks are shown in Table 4. Whilst rhyme judgement was significantly left lateralised during the fast paced presentation (*t*(17)=4.4, *p* < .001, cohen's *d*_z_=1.0) lateralisation was not significant during the slow paced task at the group level (*t*(17)=1.5, *p*=.15, cohen's *d*_z_=.35). Significant right hemisphere lateralisation was found for both the slow and the fast paced line conditions (slow *t*(17)=4.1, *p*=.001 cohen's *d*_z_=; fast *t* (17)=12.5, *p* < .001, cohen's *d*_z_=2.9). Mean blood flow plots are shown in Fig. 4. Fig. 5 shows plots of the distribution of individual LIs for each of the four conditions.

Correlations revealed no evidence for a relationship between the strength of lateralisation in the rhyme and line tasks when performed at the slow pace (*r*=−.10, *p*=.70) nor at the fast pace

##### 3.2.2.3 Assessing the effect of pace on strength of lateralisation

As in Experiment 1, we used the reversed values for line judgement LIs in order to assess the effect of pace on the strength of lateralisation. Using absolute values would obscure the fact that some participants showed left lateralised (positive) LIs during line judgement.

A 2 × 2 repeated measures ANOVA revealed a main effect of task (*F*(17)=7.07, *p*=.017, MSE=3.11) with line conditions more strongly lateralised than rhyme, and a main effect of pace (*F*(17)=9.35, *p*=.007, MSE=1.38) with stronger lateralisation in the faster conditions. The interaction was not significant (*F*=.21, *p*=.66, MSE=1.26.).

### 3.3 Summary of Experiment 2

In Experiment 2, we tested the effect of pace on blood flow lateralisation during linguistic and non-linguistic judgements. An increase in the number of judgements to be made in the active period significantly affected behavioural performance on rhyme and line judgement in both accuracy and reaction times. Increased pace negatively affected response accuracy on the line judgement task, to a greater extent than for rhyme judgement. The strength of lateralisation in both rhyme and line judgement tasks was affected by increased pace, with stronger left and right lateralisation in fast paced rhyme judgement and line judgement respectively. This was coupled with the observation that in the fast paced conditions, fewer participants were in the ‘low’ lateralised category, for both tasks.

## 4 General discussion

The two experiments reported here were designed to address methodological questions about the role of task demands, specifically stimulus presentation rate, on hemispheric lateralisation measured using fTCD. We demonstrated that lateralisation can be robustly established using two novel fTCD tasks: a language task that does not require generation of novel items, and a non-linguistic line array judgement task, which was well matched to the linguistic task in stimulus format and output requirements. By manipulating the number of stimuli presented during a trial, we also demonstrated a clear effect of task demands on lateralisation for both the linguistic and non-linguistic tasks. We will now discuss each of these findings in turn.

### 4.1 Linguistic and non-linguistic judgement tasks

Several previously published fTCD studies with adults have used tasks other than free word and sentence generation to assess the sensitivity of the fTCD technique to measure language lateralisation. For example, Badcock et al. (2011) asked participants to passively listen to a short story accompanied by pictures, the final word of which was replaced with a pure tone. it was expected that participants would implicitly generate the word to complete the sentence. In a separate task, participants were asked to listen to a definition of an object and name the object during the active period. Stroobant et al. (2009) asked participants to generate grammatically correct sentences from jumbled words, to read a fixed number of words from a text and to make self-paced semantic decisions between three visually presented words. In these studies, the language tasks led to left hemisphere lateralisation at the group level. However, in each study the average laterality indices reported were low in contrast to those recorded during word generation from the same participants. Furthermore, the proportions of individuals showing robust left lateralisation were low.

In the current study we used rhyme judgement as an alternative to word generation. Participants made button press responses to indicate whether two written word pairs rhymed. Rhyme judgement, we reasoned, does not require mental generation of new items, but still sufficiently engages articulatory planning processes. This task has been reliably shown to be left lateralised in the majority of right-handed participants as measured by the BOLD response in a number of fMRI studies (Kareken et al., 2000; Lurito et al., 2000; Pugh et al., 1996). The data from Experiment 1 showed that fTCD can indeed reliably measure changes in speed of blood flow speed associated with a nongeneration task and is sufficiently sensitive to measure the left lateralised cognitive demands of rhyme judgement, despite differences between BOLD and CBFV/rCBF (Mechelli et al., 2000).

fTCD has also been used to examine lateralisation during non-linguistic tasks such as: visual memory (Groen et at., 2011), mental rotation (Serrati et al., 2000), figure assembly, cube comparison and selecting an identical figure from an array (Bulla-Hellwig et al., 1996; Hartje et al., 1994). Whilst results from these studies have been mixed, and some showed low or no lateralised responses (Hartje et al., 1994), more recent line bisection and visual memory tasks have shown replicable and reliable right lateralisation (Rosch et al., 2012; Whitehouse and Bishop, 2009). In the current study we used line array judgement in an attempt to closely match the task demands of the rhyme judgement task. This close matching of the linguistic and non-linguistic tasks allows us to address the relationship between lateralisation for linguistic and non-linguistic skills within participants. Previous fTCD studies that have addressed this issue have not matched linguistic and non-linguistic conditions for task requirements (e.g. Dorst et al., 2008). In the current study participants made button press responses to indicate whether two sets of lines were oriented in exactly the same way or whether two words rhymed. We demonstrated, as predicted, significant left hemisphere lateralisation during rhyme judgement and right hemisphere lateralisation during line judgement. We did not observe any significant correlations between the strength of lateralisation during performance of the linguistic and non-linguistic tasks. This is not surprising given that we did not recruit left handers (who are more likely to show right lateralisation for language than right handers) and therefore could not investigate this relationship at the population level as other studies have done (Badzakova-Trajkov et al., 2010; Whitehouse and Bishop, 2008; see Cai et al. (2013) for a discussion).

### 4.2 The effect of pace of stimulus presentation on strength of laterality index

In Experiment 1, using a slow stimulus presentation rate, we found lower LI values than are typically reported in studies requiring word generation, and fewer participants than expected showing significantly lateralised blood flow. This pattern of ‘weak’ lateralisation was also observed during the line judgement task. Previous studies that have used language tasks other than word or sentence generation have attributed low lateralisation to increased right hemisphere involvement (Buchinger et al., 2000; Stroobant et al., 2011), arguing for the recruitment of distributed higher cognitive processes such as theory of mind or inference during story comprehension. Stroobant et al. (2011) also suggest that less lateralised responses during listening to stories may be due to reduced motoric demands in contrast to generation tasks. Similary, Badcock et al. (2011) attributed lower lateralisation in their receptive task to inconsistent or weaker implicit production when participants are expected to label a missing word. With regard to non-linguistic tasks, it has been argued that strong right hemisphere lateralisation is most likely to be found during tasks that combine visual attention and visuomotor manipulation and tasks that do not include both factors are likely to show weak effects (Vingerhoets and Stroobant, 1999).

In Experiment 2, we tested the hypothesis that previous linguistic and non-linguistic tasks that have shown weak lateralisation may simply not have been sufficiently demanding to drive detectable hemispheric lateralisation. Participants made (blocked) rhyme or line judgements during fast or slow presentation rates of stimulus pairs. Faster presentation, and therefore more judgements to be made within the same time window, led to higher LIs than during the slow condition. This effect of pace held for both the rhyme and line judgement tasks since there was a main effect of pace and no interaction with task type. At the individual level, twice as many participants were categorised as significantly left-lateralised for the rhyme task and right lateralised for the line task during fast presentation compared to slow presentation speeds.

It is important to emphasise that the slow and the fast paced conditions had the same stimuli and the same task requirements. It seems plausible therefore, that previous linguistic (but ‘non-generation’) tasks that have been used in the literature (e.g. reading aloud or sentence completion) were not taxing enough, or did not stimulate a sufficient degree of articulatory rehearsal in order to drive detectable left hemisphere lateralisation. For example, reading high frequency words (Stroobant and Vingerhoets, 2000) requires little phonological processing demands and articulating a single item (Badcock et al., 2011) requires negligible articulatory planning or rehearsal. Similarly, non-linguistic paradigms that have not found significant lateralisation (e.g. cube comparison and figure assembly; Bulla-Hellwig et al., 1996; Hartje et al., 1994; Serrati et al., 2000) required single responses within trials of approximately 15 s duration. These tasks may not require sufficient effort to drive detectable right hemisphere lateralisation. Our results suggest that it is not necessarily the type of task that determines the extent of lateralisation, but the effort required to complete it.

Although in the current study we found a convincing effect of increased pace, we note that the proportion of participants categorised as left lateralised during the fast rhyme task (66%), and the mean LI (1.6) were both relatively low compared to previous ‘gold standard’ word generation studies. There are a number of possible reasons for this. First, using fMRi it has been established that word generation leads to activation over a large portion of the left hemisphere in contrast to rhyme judgement, which shows more focal inferior frontal cortex activity (Lurito et al., 2000). Since fTCD measures only relative differences in blood flow speed between the hemispheres, it may be that lateralised activity in more extensive regions leads to stronger LIs than in more focal regions. Second, whether a participant is categorised as significantly lateralised (using a one sample *t*-test) depends on the number of epochs measured and the consistency of that individual's LIs over all the epochs. Some of our conditions had lower split-half reliability than has been reported in studies of word generation (e.g. Gutierrez-Sigut et al., 2015), which may have contributed to fewer participants being categorised as significantly lateralised. It is possible that consistency across trials, and hence split-half reliability, may be improved in future studies by extending the relaxation period or increasing the number of trials.

Despite weaker lateralisation during rhyme judgement in contrast to previous studies of word generation, we argue that rhyme judgement could be a valuable clinical assessment tool, since the best surgery outcomes are likely to come from the use of a battery of language tasks (Gaillard et al., 2004; Ramsey et al., 2001). Moreover, if we wish to better understand which characteristics drive the fTCD signal, externally paced tasks allow a much greater degree of experimental control, including control of number of words produced, than word or sentence generation.

Based on the findings from the non-linguistic task, and the effect of the pace manipulation on behavioural performance, we speculate that task difficulty is a driving factor in the increase in lateralised blood flow, in addition to the amount of articulatory rehearsal. If the effect of pace was related to an increase in premotor activity alone, due to greater articulatory planning, then we would expect the influence of pace on the strength of LI to be evident only in rhyme condition. However, faster pace of judgements led to increased LIs in both the linguistic and non-linguistic conditions. We therefore suggest that task difficulty does indeed play a role in lateralisation of blood flow, as measured by fTCD in the middle cerebral arteries, above and beyond articulatory rehearsal.

It is interesting to note that a previous fTCD study which manipulated task difficulty of a non-linguistic task, reported an influence of task difficulty on behaviour but not on strength of LI (Rosch et al., 2012). Participants were there required to perform a line bisection task and task difficulty was manipulated in two ways: stimulus duration and distance of stimulus from the midline. That these manipulations of ‘task difficulty’ did not influence LI but our manipulation of pace of stimulus presentation did, is perhaps not surprising. The increased effort required to solve more complex tasks versus that required for faster paced tasks would likely be mediated by different processes. Future studies with direct contrasts of such manipulations are needed to address this issue.

Although the BOLD signal and CBFV may not relate to pace in the same way (Rees et al., 1997) we can at least speculate about the areas that might drive the greater degree of hemispheric lateralisation during speeded rhyming from studies using other neuroimaging modalities. Price et al., (1996) using PET found a main effect of stimulus presentation rate during overt and covert word reading tasks in visual, motor and language related areas including left dorsolateral prefrontal cortex. Similarly, Shergill et al. (2002), using fMRI, reported that increased presentation rate, from 15 words per minute to 60 words per minute in a covert generation task, increased strength of activation in left inferior frontal gyrus, and anterior part of the left superior temporal gyrus. These areas lie within the perfusion territory of the MCA and therefore in-creased involvement of these areas is likely to affect the TCD signal.

### 4.3 A comment on categorisation

Our data demonstrate that an increase of stimulus presentation pace resulted in a higher proportion of participants being categorized as significantly ‘lateralised’: left for the rhyme task and right for the line task. A small shift in either the mean LI or standard error for an individual resulted in a change of category – left, right, or low lateralisation. We suggest that these results highlight the importance of moving away from the categorisation of participants into left, right and low groups, reserving categorical variables for discrete groups. This is not a new idea; it has long been suggested that the use of continuous variables results in greater power (Cohen, 1983; Maxwell and Delaney, 199note3, Naggara et al., 2011; Royston et al., 2006). Dichotomisation results in a loss of data, and neglects within-group variability. Using a categorisation approach, some participants may be confidently placed within a category, while data from other participants may place them on the threshold between categories. However, the category thresholds are arbitrarily defined or, more problematically, data-driven. In terms of developmental studies, test–retest reliability estimates could be misleading if a change in category is reported from a small shift in lateralisation index. As Naggara et al. (2011) note, “What is necessary or sensible in clinical and therapeutic settings in not relevant to how research data should best be analysed”.

We hope therefore to move away from the categorical distinctions of left-lateralised/low-lateralised/right-lateralised for individuals. In the absence of categorical variables it is then easier to assess repeated measures by accounting for non-independent residuals. Using general linear model type analyses (e.g. ANOVA or regression), the presence of a high proportion of low laterality indices will be accounted for. It makes little sense to exclude participants who show ‘low’ lateralisation due to an arbitrary threshold. If a participant’s LI is not statically different from zero then this will be reflected in the size of the effect. A large standard deviation of the group mean LI, along with minimum and maximum values, will indicate whether it is likely the effect is driven by one or two highly lateralised individuals. We suggest instead that examining group level trends and relationships to behaviour would be a more robust and informative way to analyse fTCD data.

### 4.4 Summary

We have demonstrated that a metalinguistic judgement task, which does not involve the overt or covert generation of novel words or sentences, can be used to assess hemispheric lateralisation of language using fTCD. We also demonstrated that a non-linguistic task, with similar task demands as rhyme judgement-line array judgement, can also be used to assess right hemisphere lateralisation.

Importantly, we demonstrated significantly greater hemispheric lateralisation when rhyme and line judgements are presented at a fast compared to a slow pace. Whilst it is tempting to attribute the stronger left hemisphere lateralisation during faster rhyme judgements to increased premotor demands alone, the finding that right hemisphere lateralisation for line judgements was also stronger for fast compared to slow paced presentation rate, suggests that general ‘task difficulty’ also plays a role in influencing the strength of laterality index. Thus we suggest that fTCD is sensitive to increased premotor demands and also to task difficulty, which may or may not be driven by a spatially distinct area within the territory of the MCAs. Future studies are needed that explicitly disambiguate the influence of these factors, for example by using fixed pace linguistic judgements of varying difficulty. In addition, manipulating the variables of pace and task difficulty separately in a non-linguistic task such as line judgement may shed light on the conflicting pattern of results between the current results and previous fTCD studies of task difficulty in spatial tasks (Rosch et al., 2012).

Our findings advance our understanding of the sensitivity of fTCD as a technique to assess hemispheric lateralisation of function. This understanding is fundamental if this technique is to be used to its full potential in providing insights into the development of hemispheric lateralisation of function in young children (Bishop, 2013).

## Appendix A. Supplementary material

Supplementary dataassociatedwiththisarticlecanbefoundin the onlineversionat http://dx.doi.org/10.1016/j.neuropsychologia.2015.04.019

Appendix A

## Figures and Tables

**Table 1:** Example word pairs for the rhyming condition.

Rhyming	Non-rhyming
Cone–sewn	Part–boot
Float–quote	Bomb–foam
Pie–sky	Pot–fly

**Table 2:** Accuracy and reaction time summaries for rhyme and line judgement tasks in Experiment 1.

Task	Accuracy (%) Mean (sd)	Reaction time (s) Mean (sd)
Rhyme	96.2 (2.9)	1.26 (.24)
Line	96.7 (2.4)	1.45 (.26)

**Table 3:** The left side of the table shows descriptive statistics of Lateralisation Indices for both conditions in Experiment 1. The right side of the table indicates the percentage of individuals who were categorised as left, right, or low lateralised.

Task	Mean (sd)	Median (interquartile range)	#Left (%)	#Right (%)	#Low (%)
Rhyme	.84 (1.80)	1.3 (−1.2–1.8)	36	14	50
Line	−1.64 (1.96)	−2.1 (−2.9 – −1.0)	7	50	43

**Table 4:** The left side of the table shows descriptive statistics of lateralisation indices (LIs) for each condition in Experiment 2. The right side of the table indicates the percentage of individuals who were categorised as left, right, or low lateralised.

Task		Mean (sd)	Median (interquartile range)	Left (%)	Right (%)	Low (%)
Rhyme	Slow	.67 (1.88)	1.19 ( −1.3 to 2.0)	34	11	55
	Fast	1.60 (1.58)	1.79 (.9–2.4)	66	6	28
Line	Slow	−1.90 (1.93)	−1.96 ( −3.1 to −1.0)	6	44	50
	Fast	−2.62 (.89)	−2.55 ( −3.6 to −1.9)	0	94	6
