# Supplementary material for: Stimulus rate increases lateralisation in linguistic and non-linguistic tasks measured by functional transcranial Doppler sonography
Source: Neuropsychologia. Author manuscript; Available in PMC 2016 Jun 27. (PMC4922413; doi:10.1016/j.neuropsychologia.2015.04.019)
Supplement: Appendix A [file NIHMS68906-supplement-Appendix_A.docx]

Appendix

Rhyme and non-rhyme pairs

Rhyming pairs

| CRY | HIGH |
| --- | --- |
| FATE | WEIGHT |
| GREW | CLUE |
| BRUISE | SNOOZE |
| HOE | SNOW |
| PHONE | KNOWN |
| GLUE | SHOE |
| WHITE | RIGHT |
| FRAIL | SCALE |
| BLOWN | STONE |
| BREAK | LAKE |
| CHEF | DEAF |
| JAIL | WHALE |
| BEER | HEAR |
| TOES | BLOWS |
| PIE | SKY |
| RULE | POOL |
| NONE | RUN |
| SPOON | JUNE |
| KITE | LIGHT |
| STUFF | TOUGH |
| FREE | TEA |
| CARE | FAIR |
| FLOAT | QUOTE |
| RARE | SWEAR |
| PEARL | GIRL |
| SOAK | JOKE |
| SIGN | LINE |
| POOR | STORE |
| ROOM | TOMB |
| CHOOSE | NEWS |
| SOME | HUM |
| CONE | SEWN |
| MOOSE | JUICE |
| FIGHT | BITE |
| THERE | HAIR |
| TRUE | FLEW |
| FADE | RAID |
| TRAIN | CANE |
| SHEET | MEAT |
| HAIL | SALE |
| FOUR | MORE |
| LOAN | BONE |
| MEET | EAT |
| CHAIR | PEAR |

Non-rhyming pairs

| COT | BUY |
| --- | --- |
| CHIP | THROUGH |
| HIDE | FOOD |
| SWERVE | PLEASE |
| BEG | KEY |
| BEAD | MAID |
| COAT | PUT |
| CARD | STAIR |
| WORK | ROAR |
| TOWED | GOOD |
| VOICE | WISE |
| DART | HATE |
| LOAD | SAID |
| SKIN | CHAIN |
| COOL | TOLL |
| POT | FLY |
| NAME | THUMB |
| FINE | DAWN |
| PLEAT | SHOOT |
| BED | KNEE |
| GUARD | FLAIR |
| COAL | BULL |
| CALM | SNAIL |
| BOMB | FOAM |
| SPEAK | FLAKE |
| SHALL | CRAWL |
| BROAD | WOOD |
| TIED | BREAD |
| LOOP | POPE |
| SOON | CROWN |
| TOOTH | PLOUGH |
| WINE | BUN |
| BOOTH | NO |
| SPILL | CRUEL |
| SHINE | LOSS |
| PART | BOOT |
| CHIN | PRUNE |
| HALF | NAIL |
| CLOCK | SPOKE |
| CHEAT | DATE |
| CART | LATE |
| YAWN | PLANE |
| FOIL | HOLE |
| MESS | NOSE |
| TERM | DREAM |
